# Supplementary material for: Vaccination with mycobacterial lipid loaded nanoparticle leads to lipid antigen persistence and memory differentiation of antigen-specific T cells
Source: bioRxiv. 2023 Jul 13:2023.03.07.531489. Originally published 2023 Mar 8. Preprint. [Version 2] doi: 10.1101/2023.03.07.531489 (PMC10028924; doi:10.1101/2023.03.07.531489)

bioRxiv preprint doi: https://doi.org/10.1101/2023.03.07.531489; this version posted July 13, 2023. The copyright holder for this preprint (which was not certified by peer review) is the author/funder, who has granted bioRxiv a license to display the preprint in perpetuity. It is made available under aCC-BY 4.0 International license.

Supplementary Figure Legends

Fig. S1. Physicochemical characterization of PLGA-NP. (A) CryoTEM image and DLS. **(B)** SAXS scattering curve fitted with a sphere model.

Fig. S2. Intracellular localization of PLGA-NP and BCN. (A) Representative confocal image of intracellular localization of PLGA or BCN (Texas red Dextran dye), lysosome (lysotracker), nucleus (NucBlue). **(B)** Quantification of co-localization of lysotracker with Texas red dye by Pearson correlation. (8 cells were analyzed per experiment, 3 independent experiments), ****p<0.0001.

Fig. S3. MA encapsulated by either BCN or MC formulation leads to lipid antigen persistence. hCD1Tg mice were IT vaccinated with MA-BCN or MA-MC at 6 weeks or 1 week prior to adoptive transfer of cell trace stained DN1 T cells. T cell activation and proliferation was measured 1 week after adoptive transfer. **(A)** Experimental diagram. **(B)** Percentage of proliferating DN1 T cells in LN, lung, and spleen. **(C)** Percentage of CD44-expressing DN1 T cells in the LN, lung, and spleen. N = 3 or 4 per condition. Data represented as mean ± SEM. ns = not significant, *p<0.05.

Fig. S4. P25-specific T cell activation and proliferation and bacterial burden in attenuated Mtb vaccination. hCD1Tg were SC vaccinated with attenuated Mtb. **(A)** Bacterial burden in the spleen and lung were determined at 2- or 6-weeks post vaccination. hCD1Tg mice were vaccinated with attenuated Mtb and after 6 weeks, CellTrace-labeled p25 T cells were adoptively transferred into the mice. After 1 week, T cell activation and proliferation was measured. **(B)** Representative FACS plots of in LN. **(C)** Percentage of proliferating P25 T cells in the LN and spleen. **(D)** Percentage of CD44-expressing P25 T cells in the LN and spleen (N = 5).

Fig. S5. Gating strategy for analyzing biodistribution of BCN in the lung. Lung single cell suspension was gated as follows: $CD45^-$ cells, neutrophils ($Ly6G^+$), alveolar macrophages

bioRxiv preprint doi: https://doi.org/10.1101/2023.03.07.531489; this version posted July 13, 2023. The copyright holder for this preprint (which was not certified by peer review) is the author/funder, who has granted bioRxiv a license to display the preprint in perpetuity. It is made available under aCC-BY 4.0 International license.

(CD11c^{+}SiglecF^{+}), DCs (CD11c^{+}), monocytes (CD11b^{+}CD11c^{-}), B cells (CD19^{+}), T cells (CD3^{+}), NK cells (NK1.1^{+}), and eosinophils (CD11c^{-}SiglecF^{+}).

Fig. S6. Representative FACS plot of alveolar macrophages within enriched and flow through fractions.

Fig. S7. Memory and naïve DN1 T cells display distinct gene expression profiles. Memory (CD44^{+}CD62L^{+}) and naïve (CD44^{-}CD62L^{+}) DN1 T cells were sorted from LNs of MA-BCN vaccinated hCD1Tg-DN1 BM chimeras at 6 weeks post vaccination and subjected to RNAseq analysis **(A)** Representative figure of CD44 and CD62L expression in DN1 T cells in LN, lung, and spleen. **(B)** MA plot representing memory vs naïve analysis. **(C)** PCA of normalized counts. N=3 per condition.

bioRxiv preprint doi: https://doi.org/10.1101/2023.03.07.531489; this version posted July 13, 2023. The copyright holder for this preprint (which was not certified by peer review) is the author/funder, who has granted bioRxiv a license to display the preprint in perpetuity. It is made available under aCC-BY 4.0 International license.

Supplementary Figure 1

A

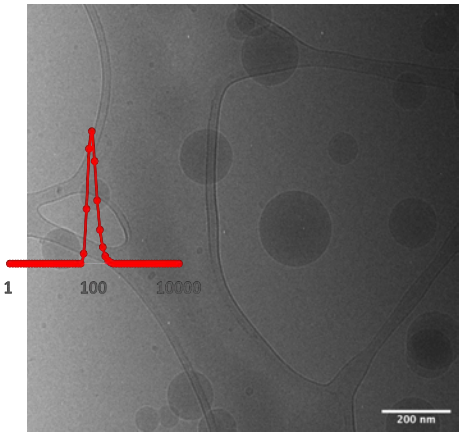

B

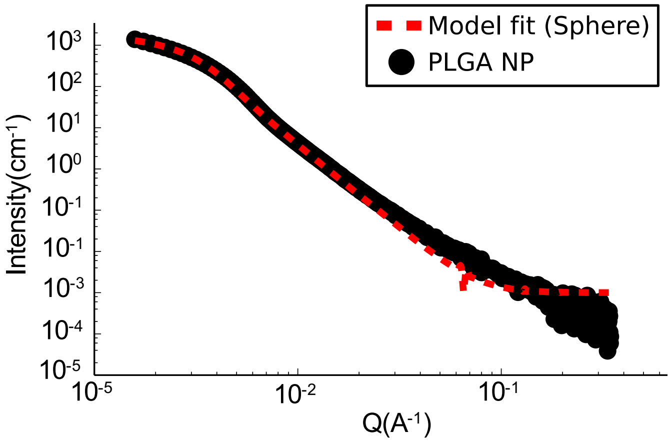

bioRxiv preprint doi: https://doi.org/10.1101/2023.03.07.531489; this version posted July 13, 2023. The copyright holder for this preprint (which was not certified by peer review) is the author/funder, who has granted bioRxiv a license to display the preprint in perpetuity. It is made available under aCC-BY 4.0 International license.

Supplementary Figure 2

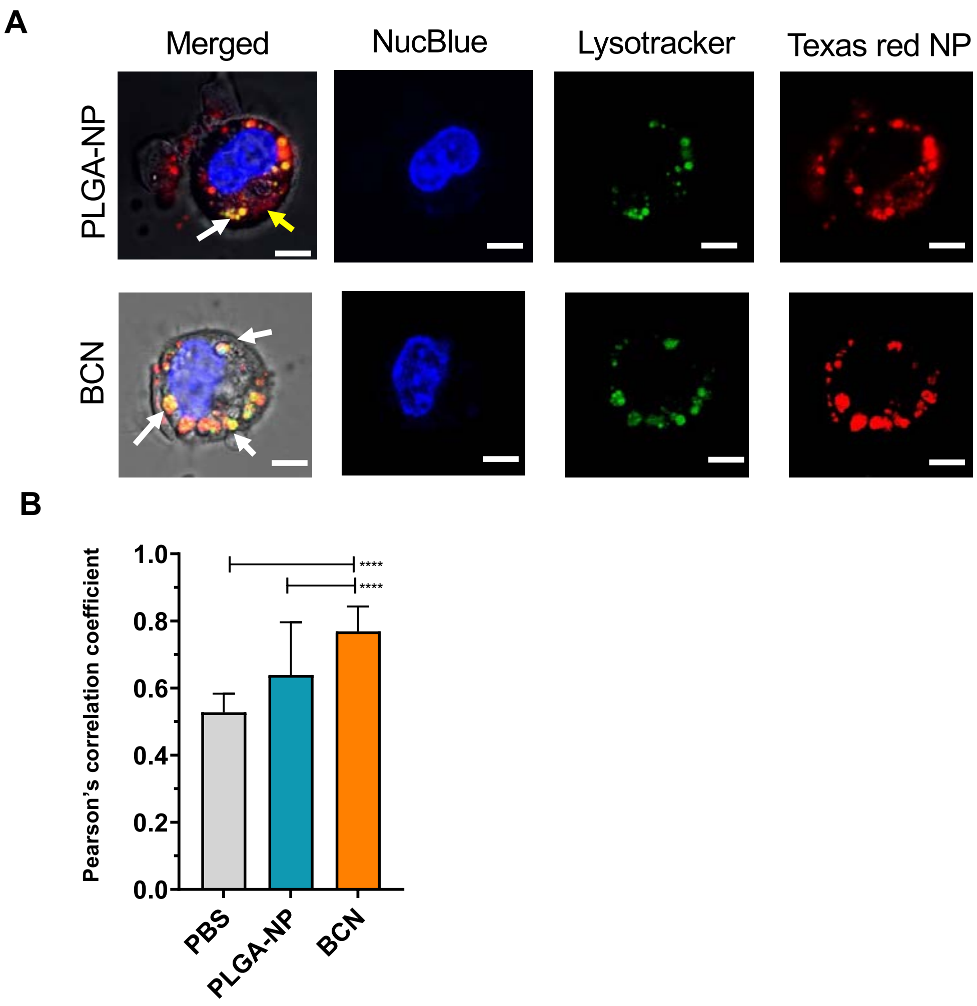

bioRxiv preprint doi: https://doi.org/10.1101/2023.03.07.531489; this version posted July 13, 2023. The copyright holder for this preprint (which was not certified by peer review) is the author/funder, who has granted bioRxiv a license to display the preprint in perpetuity. It is made available under aCC-BY 4.0 International license.

Supplementary Figure 3

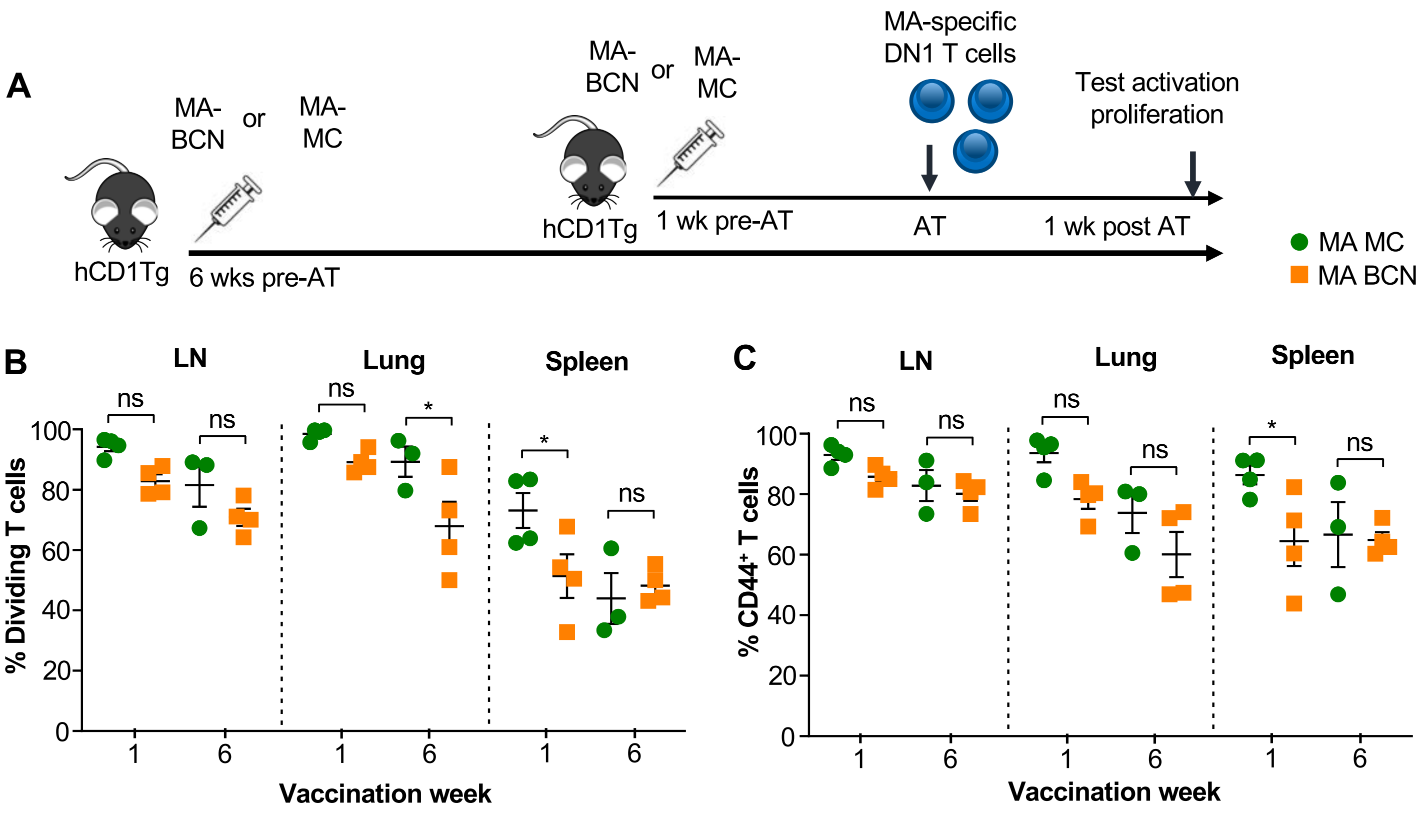

bioRxiv preprint doi: https://doi.org/10.1101/2023.03.07.531489; this version posted July 13, 2023. The copyright holder for this preprint (which was not certified by peer review) is the author/funder, who has granted bioRxiv a license to display the preprint in perpetuity. It is made available under aCC-BY 4.0 International license.

Supplementary Figure 4

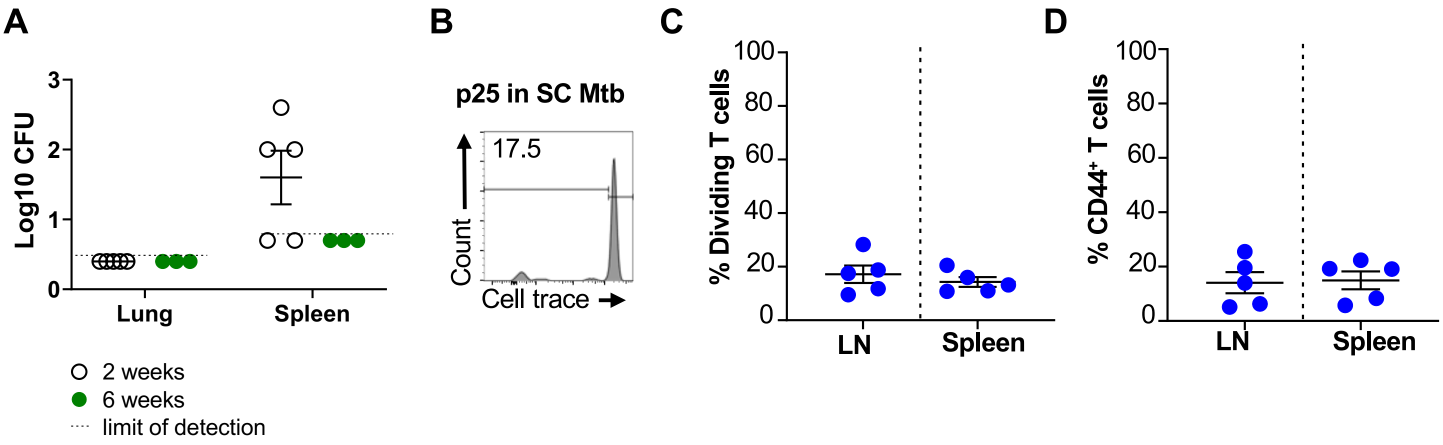

bioRxiv preprint doi: https://doi.org/10.1101/2023.03.07.531489; this version posted July 13, 2023. The copyright holder for this preprint (which was not certified by peer review) is the author/funder, who has granted bioRxiv a license to display the preprint in perpetuity. It is made available under aCC-BY 4.0 International license.

Supplementary Figure 5

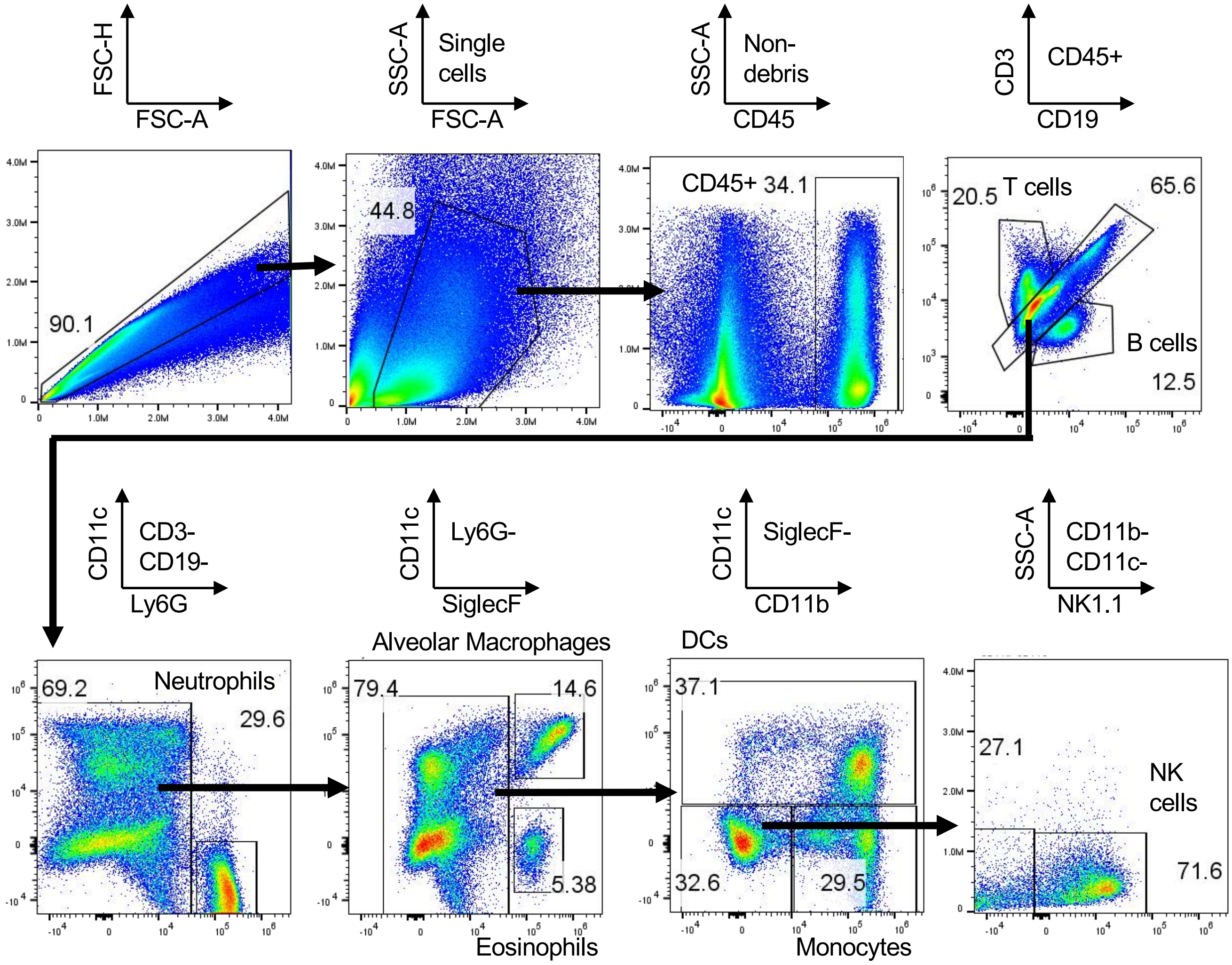

bioRxiv preprint doi: https://doi.org/10.1101/2023.03.07.531489; this version posted July 13, 2023. The copyright holder for this preprint (which was not certified by peer review) is the author/funder, who has granted bioRxiv a license to display the preprint in perpetuity. It is made available under aCC-BY 4.0 International license.

Supplementary Figure 6

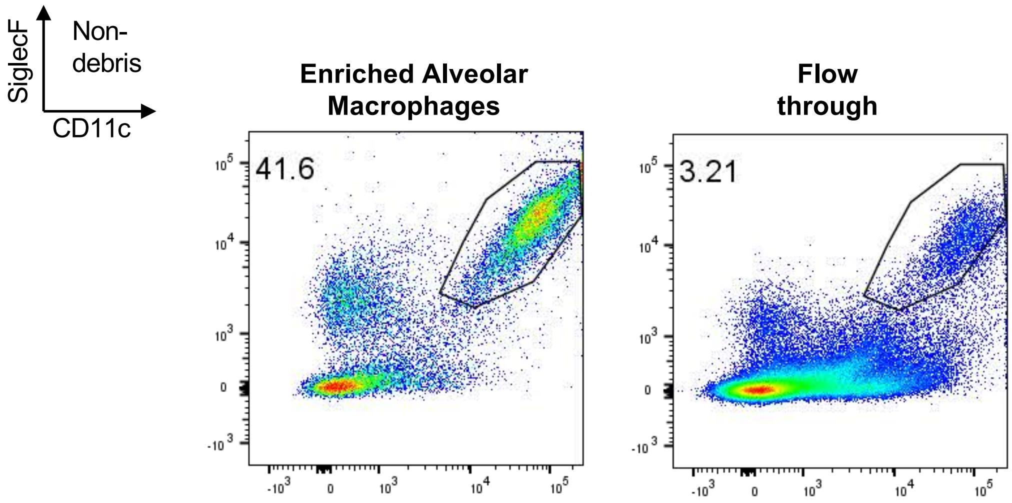

bioRxiv preprint doi: https://doi.org/10.1101/2023.03.07.531489; this version posted July 13, 2023. The copyright holder for this preprint (which was not certified by peer review) is the author/funder, who has granted bioRxiv a license to display the preprint in perpetuity. It is made available under aCC-BY 4.0 International license.

Supplementary Figure 7

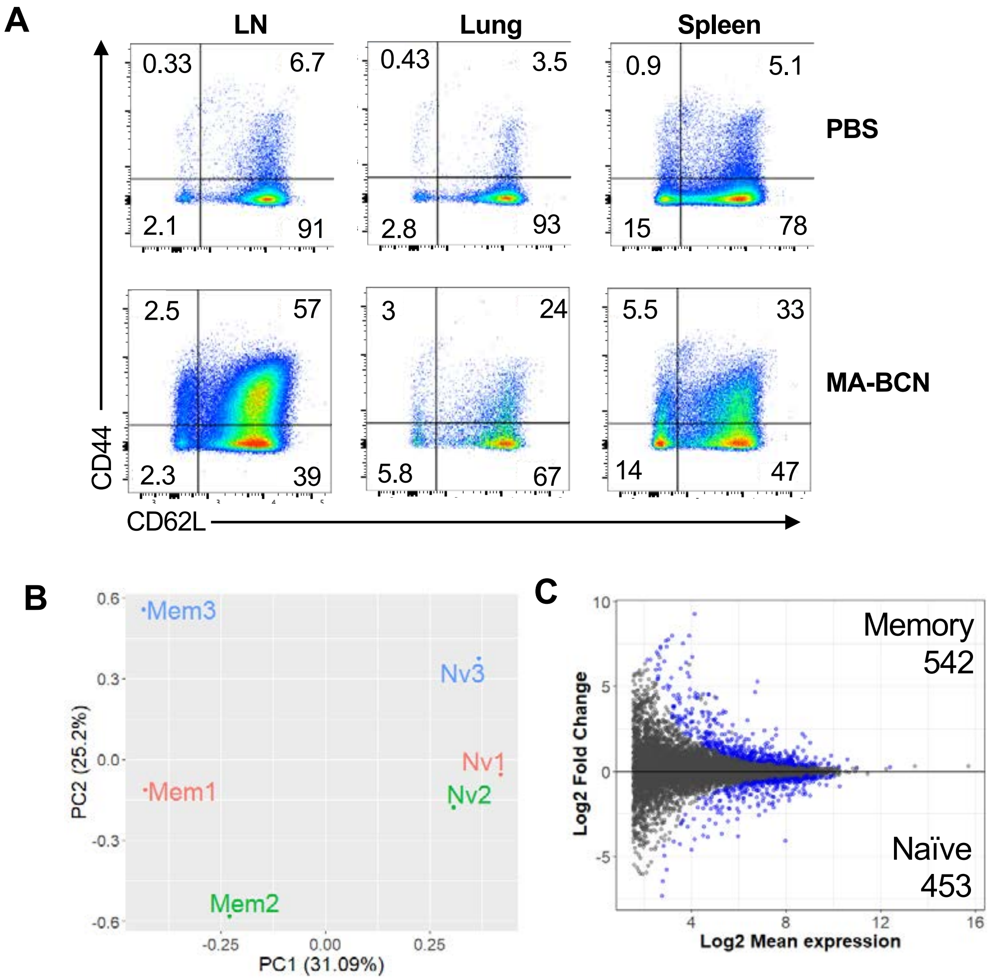

Supplement: Supplement 1 [file NIHPP2023.03.07.531489v2-supplement-1.pdf]
